# Special Issue “Zebrafish: A Model Organism for Human Health and Disease”

**DOI:** 10.3390/ijms26104624

**Published:** 2025-05-12

**Authors:** Jyotshna Kanungo

**Affiliations:** Division of Neurotoxicology, National Center for Toxicological Research, U.S. Food and Drug Administration, Jefferson, AR 72079, USA; jyotshnabala.kanungo@fda.hhs.gov

In 1996, a special zebrafish (*Danio rerio*) issue of the journal *Development*, with a 481-page volume containing 37 papers from four different laboratories, published genetic and phenotypic details of hundreds of different mutants, obtained through mutagenesis screening. The collective data showed developmental defects in almost every organ or tissue of the early life stages of zebrafish. Those findings not only revealed a wide array of information in the areas of basic science but also provided a plethora of information to investigate many human diseases. As an emergent vertebrate animal model then, zebrafish has since become the second most important animal model next to rodents, and research using the model has become a flourishing field in studying human diseases [1]. A PubMed search on “zebrafish and human disease modeling” (26 March 2025) yields a total of 1489 papers showing an exponential increase in published papers in the last two decades (Figure 1). Zebrafish share 70% of protein-coding genes with humans. One of the major advantages of using zebrafish in research is that a single female lays hundreds of transparent eggs that are externally fertilized, which helps researchers increase the sample size for their experiments. Because of the transparency of the embryos, a longitudinal assessment of developing organs and reporter transgene expressions can be easily performed by imaging with microscopes. Additionally, zebrafish can breed at around three months of age, thus becoming a cost-effective animal model for research. As an alternative animal model, zebrafish align well with the three Rs (reduction, refinement, and replacement) that are meant to address the humane use of animals. With comparable genetic and physiologic make-up between zebrafish and mammals, including humans, zebrafish possess many advantages for biomedical and toxicological research. Nearly 10 compounds from zebrafish screens conducted by several different laboratories are in or about to enter the clinic [2].

The Special Issue “Zebrafish: A Model Organism for Human Health and Disease” contains nine papers covering various aspects of pathological conditions modeled in zebrafish and the underlying mechanisms highlighting strategic interventional avenues. These studies used both wild-type and genetically modified zebrafish to address tissue/organ-specific conditions and covered a wide array of topics such as the nervous system, inflammation, cancer, and the cardiovascular system. The authors provide in-depth insight into these domains, which can lead to understanding equivalent human conditions along with predictive scenarios that may be used for future drug discovery.

In the first paper, Ciapaite et al. described the generation of the first zebrafish model of hypophosphatasia (HPP), which exhibits multiple features characteristic of the human disease, thus providing a model system for not only the pathophysiology study of HPP in depth but also the testing of compounds that have therapeutic value [3]. HPP is a rare inherited metabolic disorder that adversely affects the development of bones and teeth due to a pathogenic variation in the enzyme tissue-nonspecific alkaline phosphatase (TNSALP), encoded by the *alpl* gene [4]. The authors created the *alpl* knockout zebrafish using clustered regularly interspaced short palindromic repeat (CRISPR)/CRISPR-associated protein 9 (CRISPR/Cas9) gene editing. There was decreased bone mineralization in the *alpl^−/−^* embryos, a hallmark of HPP. These embryos had depleted pyridoxal and its degradation product 4-pyridoxic acid, suggesting disturbances in broad vitamin B6-related metabolism. This study provides novel mechanistic insight into the development of HPP.

In the second paper, Danila et al. described the differential effect of oxytocin on stimulus discrimination in albino and non-albino zebrafish [5]. The authors explored the potential of oxytocin in reducing psychosocial difficulties, such as anxiety, stress, stigmatization, and social isolation, including factors that affect the development and maintenance of interpersonal relationships, behaviors exhibited by individuals with albinism. The major goal of the study was to investigate how oxytocin can influence behavior, thus leading to an insight into interventional avenues and managing the negative effects associated with albinism [6]. The study used behavioral analyses of adult albino and non-albino zebrafish and concluded that oxytocin enhanced social interactions in albino zebrafish, suggesting a potential neurochemical mechanism. The authors propose that their study provides an encouraging starting point for future research that would have applicability to humans with similar behavioral disorders.

The third paper by Li et al. revealed the mechanism of how the deletion of *Slc1a4* suppresses axon regeneration in zebrafish [7]. Zebrafish are well known for their remarkable regenerative capacity [8]. In this study, the authors used *Slc1a4^−/−^* mutants created by the CRISPR-Cas9 genome editing and investigated Mauthner cell axon regeneration potential in those mutants. Since there is evidence that amino acids play a vital role in axon regeneration [reviewed in [9]], the study utilized the deletion of *Slc1a4*, an amino acid transporter that is involved in the uptake of amino acids. Although Slc1a4 deficiency did not affect Mauthner cell development or motor function, it suppressed Mauthner cell axon regeneration and negatively affected the associated functions. RNA-Seq analyses revealed that Slc1a4 may influence Mauthner cell axon regeneration via the p53 signaling pathway. The study also showed that Slc1a4 deficiency may inhibit Mauthner cell axon regeneration thorough suppression of Gap43 (Growth-Associated Protein 43), a marker and a critical factor of axonal growth and regeneration [reviewed in [10]].

Ricarte et al. [11] undertook an integrative approach that included behavioral analysis and neurotransmitter profiling to determine the effects of early-life exposure to valproic acid (VPA) both in the larval and adult zebrafish. Their study was intended to gain further insight into autism spectrum disorder (ASD) since prenatal exposure to VPA has been linked to an increased incidence of autism [reviewed in [12]]. The study showed that VPA-treated larvae exhibited hyperactivity and compromised visual and vibrational escape responses. The larvae had altered neurotransmitter levels showing increased glutamate but reduced acetylcholine and norepinephrine levels. VPA-treated embryos upon reaching adulthood exhibited larger shoal sizes and a decreased interest for their conspecifics, hallmarks of impaired social behavior in zebrafish. Their brains had significantly reduced dopamine and gamma-aminobutyric acid (GABA) levels. Data from this study’s combined behavioral and biochemical approach validate zebrafish as an ideal model of ASD.

The zebrafish has emerged as a powerful vertebrate model for studying cancer [reviewed in [13]]. Mutations in the succinate dehydrogenase subunit B (SDHB) gene are associated with the highest malignancy rate [14]. In their paper, Miltenburg et al. present a zebrafish model to study SDHB-associated phaeochromocytomas and paragangliomas (PPGLs) [15]. Their study revealed that although adult SDHB mutant zebrafish did not show an obvious tumor phenotype and were anatomically and histologically not different from their wild-type counterparts, they had significantly increased succinate levels, a characteristic of SDHB-related PPGLs. The authors conclude that they characterized an adult in vivo zebrafish model that can be used to gain in-depth knowledge on the mechanism underlying the development of SDHB-associated PPGL pathology.

The angiotensin-converting enzyme (ACE) plays a crucial role in blood pressure regulation by converting angiotensin I to angiotensin II, a vasoconstrictor that narrows blood vessels, resulting in increased blood pressure [16]. A potential link between ACE deficiency and gastrointestinal tract inflammation was revealed when several studies showed compromised proliferation and apoptosis of intestinal epithelial cells in ACE-deficient mice [17,18,19]. In their paper, Wei et al. modeled intestinal inflammation by creating ACE-deficient zebrafish using the CRISPR/Cas9 approach [20]. The authors show that the deletion of *ACE* in zebrafish not only caused intestinal inflammation but also increased the expression of several inflammation marker genes. Histopathological data revealed significantly increased secretion of mucus in the intestines of the *ACE* mutants compared to their wild-type counterparts. The mutant zebrafish showed increased susceptibility to enteritis. The authors conclude that such findings validate the zebrafish model to study intestinal inflammation and gain insight into strategies for potential therapeutic interventions.

In their manuscript on zebrafish olfactory bulb injury and recovery, Rozofsky et al. [21] show that there is remarkable plasticity of adult dendritic arbor structures following injury, which reaffirmed the existing knowledge that zebrafish is an ideal model system to study nervous system injury and neuroregenerative processes. In an earlier study, their laboratory showed that deafferentation (loss or reduction in sensory input to the central nervous system) of the olfactory bulb altered mitral cell dendritic arbor morphology [22]. As a continuation of that investigation, the current study [21] reveals that mitral cell dendritic branch regeneration and growth was independent of overall growth-related changes. The adult zebrafish model provided a system to investigate both degeneration and subsequent regeneration of the mitral cell dendritic branch in the olfactory bulb. The authors suggest that due to a lack of sufficient information on the effects of deafferentation and the recovery of aspinous dendrites in adult brain structures, their work on the zebrafish model will help fill the knowledge gap and serve as a step toward an in-depth understanding of the processes involved in regeneration following specific injuries.

In a review paper, Yang et al. [23] provide a comprehensive report on the zebrafish congenital heart disease (CHD) models that are used in research to understand and reveal the mechanisms of CHDs. The findings from such studies offer potential strategies for the treatment and intervention of CHDs. The review also outlines the advantages and disadvantages of using the zebrafish model in CHD investigation. For example, while zebrafish and the human heart share a similar electrophysiology, the zebrafish heart does not have pulmonary circulation in the double-chambered heart due to the absence of lungs. Nonetheless, the zebrafish model has been successfully used in CHD investigations, leading to revelations of the mechanisms and filling in the knowledge gap.

Lastly, a review article by Gu and Kanungo [24] details how arsenic-induced supernumerary motor neurons in zebrafish, possibly through the sonic hedgehog (Shh) pathway [25], presents an opportunity to investigate ASD. The review summarized existing reports on potential cross talk among Shh signaling, cyclin-dependent kinase 5 (Cdk5) signaling, and mindbomb (Mib) in generating excess neurons. Generation of a specific type of neurons in excess can adversely affect the development of other types of neurons. Ultimately, this will negatively affect proper development of the nervous system, otherwise known as nervous system patterning, and cause excitation/inhibition imbalance, a hallmark of ASD [26]. While Shh is obligatory for motor neuron development [27], Cdk5 is predominantly expressed in neurons and plays a crucial role in neuron development and many neuronal functions [28]. Furthermore, *Mib1* has been identified as an autism risk gene [29]. The review article proposes a potential link among these molecules (Shh, Cdk5, and Mib) in the development of ASD.

In conclusion, this Special Issue presents a collection of seven original research papers and two review articles highlighting the successful use of the zebrafish model to investigate human health-related issues and diseases. The papers cover a range of topics, from cancer, neurobehavior, and regeneration, to chemical toxicity and various state-of-the-art techniques, including the CRISPR/Cas9 gene editing technology, providing greater insights on the respective research areas. Given the increasing popularity of zebrafish as an alternative animal model in biomedical and toxicological research, this Special Issue will help expand the understanding of the underlying mechanisms of specific human diseases and health-related issues.

## Figures and Tables

**Figure 1:**
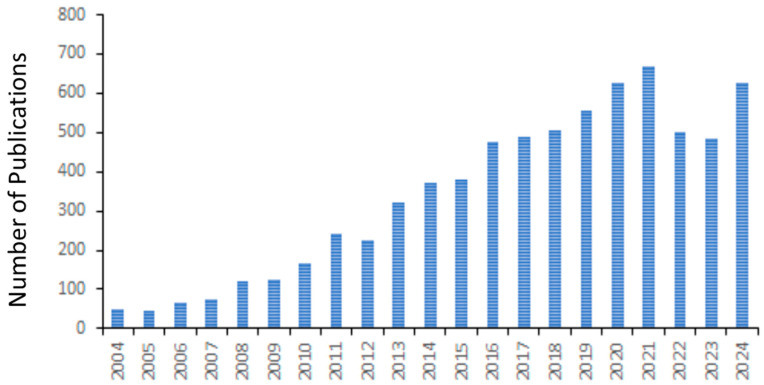
Number of publications on “Zebrafish and Human Disease Modeling” from 2004 to 2024.
